# A unique cluster of *roo* insertions in the promoter region of a stress response gene in *Drosophila melanogaster*

**DOI:** 10.1186/s13100-019-0152-9

**Published:** 2019-03-13

**Authors:** Miriam Merenciano, Camillo Iacometti, Josefa González

**Affiliations:** 1 Institute of Evolutionary Biology (CSIC-Universitat Pompeu Fabra), Passeig Maritim de la Barceloneta 37,49, 08003 Barcelona, Spain; 2 Department of Life Sciences and Systems Biology, University of Turin, Turin, Italy

**Keywords:** Transposable element, Fecundity, Viability, Target site duplication, Recurrent insertion, Natural population

## Abstract

Transposable elements (TEs) are not randomly distributed in the genome. A genome-wide analysis of the *D. melanogaster* genome found that differences in TE density across 50 kb genomic regions was due both to transposition and duplication. At smaller genomic scales, promoter regions of *hsp* genes and the promoter region of *CG18446* have been shown to accumulate TE insertions. In this work, we have further analyzed the promoter region of *CG18446*. We screened 218 strains collected in 15 natural populations, and we found that the *CG18446* promoter region contains 20 independent *roo* insertions. Based on phylogenetic analysis, we suggest that the presence of multiple *roo* insertions in this region is likely to be the result of several bursts of transposition. Moreover, we found that the *roo* insertional cluster in the *CG18446* promoter region is unique: no other promoter region in the genome contains a similar number of *roo* insertions. We found that, similar to *hsp* gene promoters, chromatin accessibility could be one of the factors explaining the recurrent insertions of *roo* elements in *CG18446* promoter region.

**Electronic supplementary material:**

The online version of this article (10.1186/s13100-019-0152-9) contains supplementary material, which is available to authorized users.

## Background

Recurrent insertion of transposable elements in specific genomic regions has been described in the *Drosophila melanogaster* reference genome. The analysis of 50 kb genomic windows identified 23 regions with a high density of TE insertions, most of them located in pericentromeric regions or on chromosome 4 [1]. Transposition and duplication were identified as the two mechanisms generating these high-density TE regions. In recent years, computational pipelines have been developed to analyze the TE content in multiple strains [2–4]. Thus, besides TEs annotated in the reference genome, non-reference TE insertions can now also be analyzed. Based on these population analyses, some genes have also been reported to accumulate many TE insertions, such as the 106.5 kb *klarsicht*, and the 24 kb *derailed-2* that were analyzed in 146 strains of the Drosophila Synthetic Population Resource [5, 6]. At a much finer scale, several insertions in the proximal promoter regions of *hsp* genes have been reported [7, 8]. While the vast majority of these insertions were *P-elements*, insertions from the *Gypsy* and the *Jockey* family were also identified. *P-elements* have a preference to insert in 5′ gene flanking regions [9]. The accumulation of TEs in the promoter of *hsp* genes was explained by the chromatin conformation of this particular region, and by selection favoring the retention of TEs because of their effect on gene expression [8]. More recently, nine *roo* insertions were also described in the promoter region of another stress response gene, *CG18446* that encodes a nucleic acid binding protein [10]. *CG18446* is a cold resistance candidate gene [11] and an ethanol-regulated gene [12] highly expressed in ovaries and in 6–10 h-old embryos [13]. Only one of the nine identified insertions was found to consistently affect the expression of *CG18446*, and it was associated with increased viability in nonstress and cold-stress conditions [10]. However, only 39 strains from two natural populations were screened, and thus it is still an open question whether more *roo* insertions are present in the *CG18446* promoter region. Indeed, *roo* are the most abundant elements in the *D. melanogaster* genome [14, 15]. Thus, it is possible that besides the cluster identified by Merenciano et al. (2016) [10] other similar clusters of *roo* insertions in gene promoter regions are present in the genome. Interestingly, while the majority of strains analyzed so far contain a *roo* insertion (26 out of 39), none of them contains more than one insertion [10].

In this work, we looked for TE insertions in the *CG18446* promoter region in 218 strains from 15 natural populations in Europe, North America, and Africa. In addition, based on the analysis of the reference genome, and on the analysis of 177 DGRP strains, we identified 53 promoter regions that could potentially contain multiple *roo* insertions. Finally, we performed fecundity and viability experiments to investigate why we did not find any fly containing two *roo* insertions in the *CG18446* promoter region.

## Results

### Twenty *roo* solo LTR insertions are present in the *CG18446* promoter region in natural populations

**Fig. 1:**
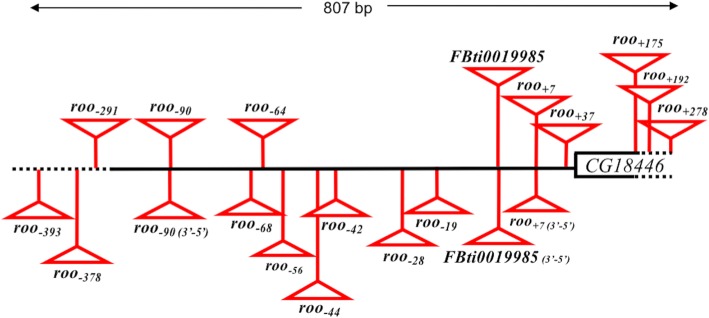
Twenty *roo* solo-LTR insertions are located in the promoter region of *CG18446* gene in different natural strains. Schematic representation of the *CG18446* promoter region where the 20 *roo* solo-LTRs are inserted. The black line represents the *CG18446* promoter region. Note that although only one insertion was found in any given strain, we have represented them together for simplicity. The white box represents *CG18446* 5’UTR. Regions depicted with dotted lines are not drawn to scale. Insertions present in 5′-3′ orientation are shown above the black line and insertions present in 3′-5′ orientation are shown below the black line

To check whether there were more *roo* insertions in the *CG18446* promoter region, we performed a PCR screening in 218 strains from 15 natural populations: 13 European, one North American [16], and one African population collected in the ancestral range of the species (Zambia) (Additional file 1) [17]. 143 strains gave a band consistent with the presence of an insertion, in homozygous or heterozygous state, and 75 strains gave a band consistent with the absence of an insertion (Table 1 and Additional file 2A). We sequenced all the obtained PCR bands and we found that besides the nine insertions discovered in Merenciano et al. (2016) [10], there are 11 other 428 bp *roo* solo-LTR insertions in the promoter region of *CG18446* (Fig. 1). All the strains with an insertion contained a single *roo* insertion. Across strains, three of the insertion sites contained *roo* elements inserted in opposite orientations, *roo*_*− 90*_, *FBti0019985* and *roo*_*+ 7*_, suggesting recurrent insertion in the same exact genomic position (Fig. 1). Recurrent insertion in the same exact genomic position has also been described for *P-elements* in *D. melanogaster* [18, 19]*.* Note that based on the results of *T-lex2* [20], a computational pipeline that estimates presence/absence of insertions based on next generation sequencing data, we previously reported that the first nine *roo* insertions described in the *CG18446* promoter region were present in Zambia [10]. However, PCR analyses of 23 of the 42 strains analyzed with *T-lex2* containing four of these nine insertions indicated that these four insertions are not actually present in any of the strains (Additional file 2B). These four unvalidated insertions were polymorphic according to *T-lex2.* Thus, it could be that these insertions have been lost in the isofemale strains since they were originally sequenced. Errors in genotyping of *T-lex2* could also explain some of these discrepancies, although all the homozygous insertions that *T-lex2* predicted were validated by PCR (Additional file 2B).

**Table 1:** PCR results and *roo* insertions identified in the 218 strains analyzed in this work, and in brackets insertions identified in the 39 strains analyzed in Merenciano et al. (2016) (10)

Population	Strains analyzed	Strains homozygous for the presence of a *roo* insertion	Strains heterozygous for the presence of a *roo* insertion	*roo* insertions identified
Akka, FI	13	3	4	*roo*_*− 90*_ *, roo*_*−64*_ *, roo*_*− 291*_
Stockholm, SE	23	9	6	*roo*_*−44*_ *, roo*_*− 68*_ *, roo*_*− 90*_ *, roo*_*− 393*_ *, roo*_*− 64*_ *, roo*_*−42*_ *, FBti0019985*_*(3′-5′)*_
Lund, SE	6	3	1	*roo*_*−68*_ *, roo*_*− 64*_
Karensminde, DK	12	5	2	*FBti0019985, roo*_*− 19*_ *, roo*_*−68*_ *, roo*_*− 64*_ *, roo*_*-90(3′-5′)*_
Munich, DE	14	6	5	*roo*_*+ 175*_ *, roo*_*−68*_ *, roo*_*− 90*_ *, roo*_*− 378*_
Market Harborough, UK	20	5	7	*FBti0019985, roo*_*+ 37*_ *, roo*_*− 68*_ *, roo*_*− 90*_ *, roo*_*− 291*_ *, roo*_*− 42*_ *, roo*_*-90(3′-5′)*_
Gotheron, FR	13	3	2	*roo*_*− 68*_ *, roo*_*− 90*_ *, roo*_*− 378*_ *, roo*_*− 64*_
Bari, IT	(12)	(3)	(4)	*FBti0019985, roo*_*+ 175*_ *, roo*_*− 19*_ *, roo*_*− 28*_ *, roo*_*− 68*_ *, roo*_*− 90*_
Gimenells, ES	14	3	9	*FBti0019985, roo*_*+ 175*_ *, roo*_*− 44*_ *, roo*_*− 90*_
Tomelloso, ES	15	3	10	*roo*_*−44*_ *, roo*_*− 90*_ *, roo*_*− 291*_
Cortes de Baza, ES	13	0	9	*roo*_*−44*_ *, roo*_*− 90*_ *, roo*_*-90(3′-5′)*_
Guadix, ES	14	0	11	*roo*_*−68*_ *, roo*_*− 90*_ *, roo*_*− 64*_ *, FBti0019985*_*(3′-5′)*_
San Cristóbal de la Laguna, ES	12	6	2	*FBti0019985, roo*_*− 90*_ *, roo*_*− 291*_
Raleigh, US	22 (27)	17 (19)	0	*FBti0019985, roo*_*+ 7*_ *, roo*_*+ 278*_ *, roo*_*− 28*_ *, roo*_*− 44*_ *, roo*_*− 68*_ *, roo*_*− 90*_ *, FBti0019985*_*(3′-5′)*_
Siavonga, ZI	27	2	10	*roo*_*− 90*_ *, roo*_*+ 7(3′-5′)*_ *, roo*_*− 56*_ *, roo*_*+ 192*_
TOTAL	257	87	82	20 *roo* insertions

**Fig. 2:**
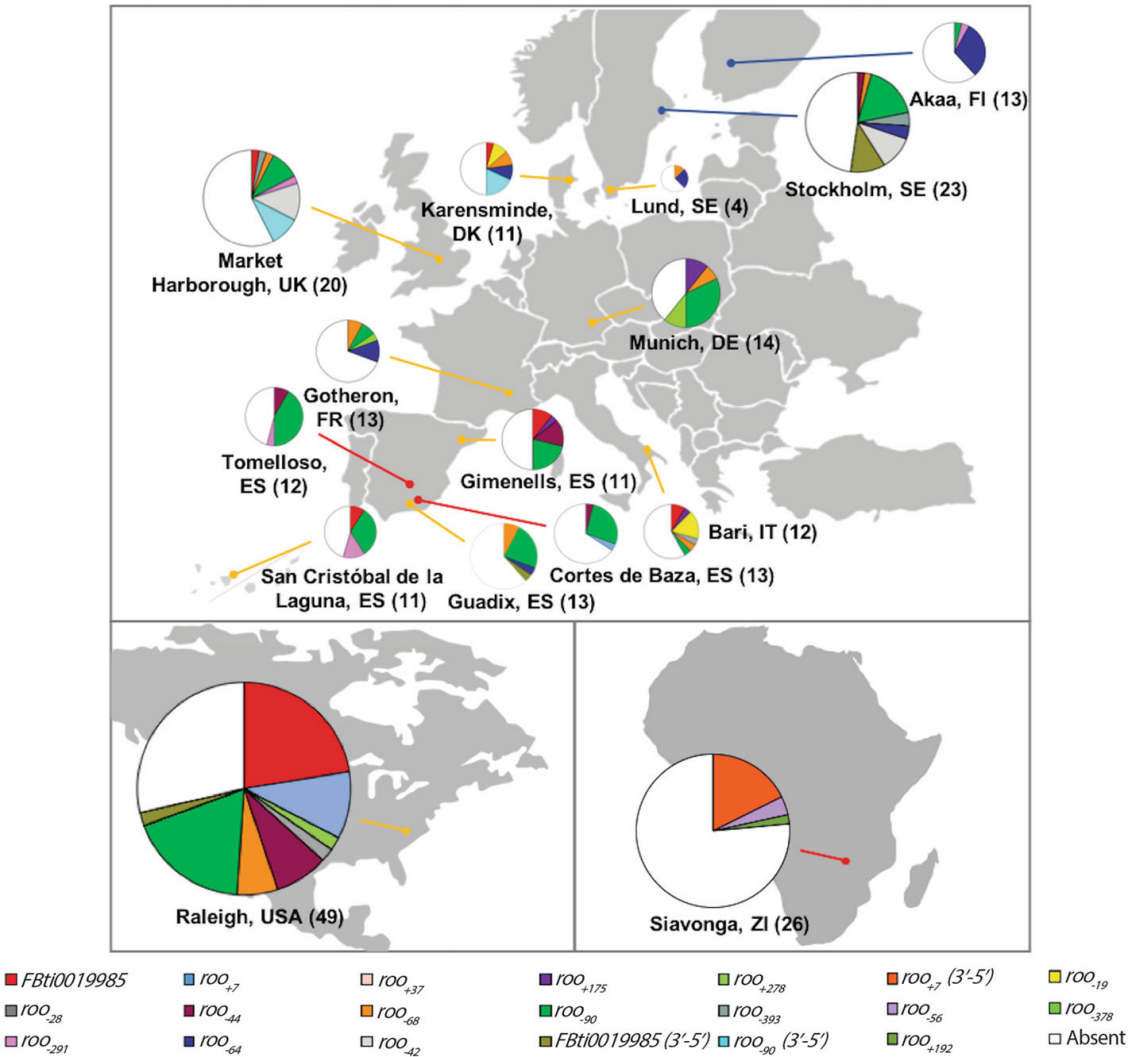
The majority of the insertions in the *CG18446* promoter region are present at very low allelic frequencies. Allelic frequencies of the 20 *roo* solo-LTRs insertions in populations from Europe, North America, and Africa. The number of strains analyzed in each population is given in parenthesis and the pie chart size is proportional to this number. Blue, yellow, and red lines represent populations with cold, temperate, and arid climates, respectively

The majority of the 20 *roo* insertions inserted in the *CG18446* promoter region were present at very low allelic frequencies, ranging from 0.2% to 16.5% (Fig. 2, Additional file 2C). The two most common insertions were *roo*_*− 90*_ and *FBti0019985*, with allelic frequencies of 16.5% and 6.3%, respectively (Fig. 2, Additional file 2C). While seven of the insertions were private, *roo*_*− 68*_ and *roo*_*− 90*_ were present in nine and 13 out of the 15 populations analyzed, respectively (Additional file 2D). We tested whether European populations at different latitudes differed in the diversity of *roo* insertions or in the total number of strains containing an insertion. Note that we did not considered the strains from Lund (Sweden) as only four strains were analyzed in this population. We found no correlation between latitude and the number of different *roo* insertions (Pearson r^2^ = 0.006, *p*-*value* = 0.793), or between latitude and the number of strains with an insertion (Pearson r^2^ = 0.063, *p-value* = 0.388). We also analyzed whether any of the insertions were more frequent in cold, temperate, or arid climates (Additional file 1). We found that *roo*_*− 90*_ was more frequent in arid climates (*p-value* < 0.001) and *roo*_*− 64*_ was more frequent in cold climates (*p-value* = 0.003) (Fig. 2).

Overall, we identified 20 *roo* insertions in the *CG18446* promoter region, most of them present at low population frequencies. While the majority of strains (169 out of 257) had one of the 20 *roo* insertions, none of the strains analyzed contained more than one *roo* insertion.

### Recurrent insertion is the most likely explanation for the presence of 20 insertions in the promoter region of *CG18446*

We identified the target site duplication (TSD) for 17 of the 20 *roo* insertions located in the *CG18446* promoter region. These 17 *roo* solo-LTR insertions have different TSDs suggesting that they are independent insertion events (Additional file 3). 15 of the 17 identified TSD were five bp-long and the consensus TSD was similar to the one previously described [10, 20, 21] (Additional file 3). Thus, multiple insertions in the *CG18446* promoter region are likely the result of transposition rather than small rearrangements such as duplications or inversions, which would change the location of the insertions but not the TSDs.

**Fig. 3:**
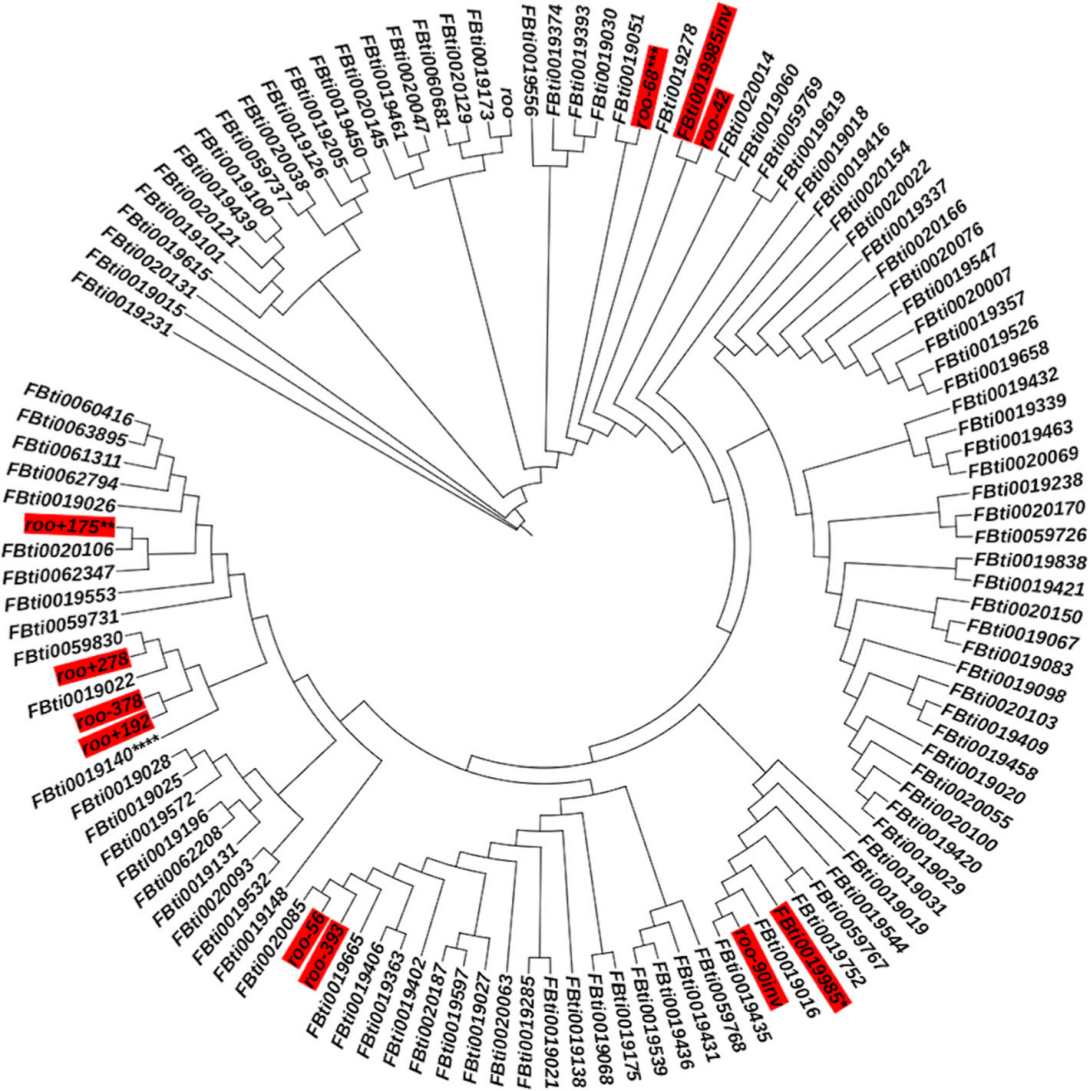
The 20 *roo* solo-LTR insertions found in *CG18446* promoter region are the result of several bursts of transposition. Phylogenetic tree including the 20 *roo* solo-LTR insertions found in *CG18446* promoter region and 115 other *roo* insertions annotated in the reference genome. *roo* solo-LTRs found in this work are highlighted in red. When several TEs with the same exact sequence were identified, we removed all of them but one. The TEs included in the tree are marked with *. The TEs that were eliminated are: (*) *FBti0019017, FBti0019394, FBti0019438, FBti0020009, FBti0020080, roo*_*+ 7*_*, roo*_*− 90*_*, roo*_*− 19*_*, roo*_*− 28*_*, roo*_*− 64*_*, roo*_*+ 37*_*, and roo*_*+7inv*_, (**) *roo*_*− 291*_*,* (***) *roo*_*− 44*_*,* and (****) *FBti0019608*

We tested whether the multiple insertions could have been the results of a burst of transposition. We constructed a phylogenetic tree for the *roo* insertions present in the reference genome, and the 20 *roo* insertions found in the *CG18446* promoter region (see Material and Methods). Briefly, we estimated the unique number of substitutions shared between the two closest TEs assuming that all the *roo* copies present in the genome derived from a common ancestral sequence [22]. We found four groups of *roo* copies that are identical to each other and thus appeared to be the result of several bursts of transposition (Fig. 3, see Material and Methods). This is consistent with *roo* being one of the most active families in the *D. melanogaster* genome [14, 15, 23, 24].

We then checked whether *roo* elements have a preference for inserting in 5′ gene regions. We considered as a 5′ gene region the 1 kb upstream of a gene and its 5’UTR region. Considering not only the 138 *roo* insertions annotated in the reference genome but also the 12,745 *roo* *de novo* insertions found in 177 DGRP strains by TIDAL software [15], we found that only 4.5% (586) of the *roo* elements are inserted in gene promoter regions or/and 5’UTR regions (see Material and Methods). This percentage is smaller than the one found for other TE families with preference for inserting in 5′ gene regions, such as the *P-element* family for which this percentage is > 77% [9, 10]. Thus, we considered that *roo* elements do not have a preference for inserting in 5′ gene regions.

We also checked whether the promoter region of *CG18446* has similarities with the promoter of *hsp* genes that could explain the high number of insertions in this region [8]. We found that, similar to *hsp* genes, *CG18446* is regulated by polymerase pausing [25], and has a high germline transcription activity [13]. Thus, chromatin accessibility could be one of the factors explaining the high TE density in the *CG18446* promoter region.

Finally, we found that transcription factor binding sites, core promoter motifs, and Matrix Associated Regions (MARs) previously described in the *roo* family were highly conserved in all the *roo* sequences described in this work (Additional file 4) [10, 26, 27].

Overall, we found that the presence of the 20 *roo* insertions in the *CG18446* promoter region is likely to be the result of several bursts of transposition (Fig. 3). Thus, recurrent insertions seem the most likely explanation for the presence of *roo* elements in the *CG18446* promoter region. Similar to the cluster of *P-element* insertions in the promoter of *hsp* genes, *roo* elements are also inserted in a promoter region with an open chromatin architecture [8].

### The *roo* insertion cluster in *CG18446* is unique

We tested whether other *roo* clusters in gene promoter regions were present in the reference genome. Out of the 137 other *roo* elements present in the reference genome, 26 are inserted in promoters (less than 1 kb from a gene) or in 5’UTR regions. These 26 *roo* elements are inserted in 26 different promoter regions, and five of them are solo LTRs (Additional files 5 and 6A). We screened by PCR the presence/absence of insertions in these 26 promoter regions in 10 randomly chosen DGRP strains (see Material and Methods). For 22 of the 26 promoter regions, no other insertion was found in any of the 10 strains. The other four promoter regions contained the same *roo* element present in the reference genome in all the 10 strains analyzed (Additional files 5 and 6A). These results suggest that considering all the *roo* insertions annotated in the reference genome only the *CG18446* promoter region contain a cluster of *roo* insertions.

Besides the *roo* insertions annotated in the reference genome, we also analyzed all the *de novo* *roo* insertions identified by TIDAL in a set of 177 DGRP strains [15, 16]. There are 559 *roo* elements inserted in promoters or in 5’UTR regions. These 559 *roo* elements are distributed in 421 gene promoter or 5′ UTR regions (Additional file 5). According to TIDAL, the promoter region of *CG18446* has a *roo* insertion in eight different DGRP strains. We focused on the 27 gene promoter regions where TIDAL identifies three or more strains containing a *roo* insertion (Additional files 5, 6B and C). In order to test whether any of the 27 promoter regions harbors different *roo* insertions, we checked by PCR and sequenced the obtained bands of the 27 gene promoters in 95 strains (Additional file 6B). Among the 27 promoter regions analyzed, only four have two different *roo* insertions in different strains (Table 2). For these four genomic regions, we performed further PCR analysis in another 10 randomly chosen strains. We could not detect any other *roo* insertion in these promoter regions, suggesting that they probably harbor only the two *de novo* *roo* insertions found before.

**Table 2:** *de novo* *roo* insertions found in four gene promoter regions

Promoter region	Number of strains predicted to have an insertion	Genomic coordinates of the insertion sites validated
*plum*	4	3R: 25,621,076 and 26,521,553
*CG11459*	3	3R: 6,027,532 and 6,027,608
*CG15879*	3	3 L: 2,169,152 and 2,169,162
*CR44657*	3	X: 14,114,700 and 14,115,661

Finally, it could be that *roo* insertions tend to form clusters, but that these clusters are deleterious when located in promoter regions. We thus also checked whether *roo* elements cluster in 1 kb regions genome-wide, not necessarily located in gene promoters. We found five 1 kb regions with seven or more *de novo* *roo* insertions located in chromosomes 2 and 3 (Additional file 7, 8A and B). Because TIDAL does not predict the exact insertion site but rather provides a range of nucleotides where the TE is inserted, it is likely that the total number of *roo* insertions predicted in these windows is an overestimate. Indeed, the two regions with more *roo* insertions, 17 and 13 insertions, overlapped 911 bp and 323 bp respectively with the *roo* cluster in *CG18446* promoter region. Based on the screening reported in this work, we know that there are eight and one insertions respectively in these two regions. We checked by PCR whether all the elements predicted within the five 1 kb regions with more than seven insertions, and two randomly chosen windows with six and four predicted insertions had the same insertion site or not. The two regions overlapping with the *CG18446* promoter region contained five and one insertion (Additional file 8A). The other five regions analyzed contained at most two *roo* insertions (Additional file 8A). Thus, we found that only the 1 kb region that overlaps with the *CG18446* promoter region is actually a *roo* insertional cluster (Additional file 8A).

### Flies with two *roo* insertions in the *CG18446* promoter regions are viable and show similar fecundity rates as flies with one *roo* insertion

As mentioned above, none of the 257 strains analyzed contains more than one *roo* insertion in the *CG18446* promoter region. The two *roo* insertions that are present at higher population frequencies are *FBti0019985* and *roo*_*− 90*_. Thus, for these two insertions, and depending on the population analyzed, we would expect to find from 0.6% to 8.8% of flies containing these two insertions in different haplotypes (Additional file 2E). Since the number of strains sampled per population is not very high (Additional file 1), it could be that we have not screened enough flies to find one strain containing two insertions.

**Fig. 4:**
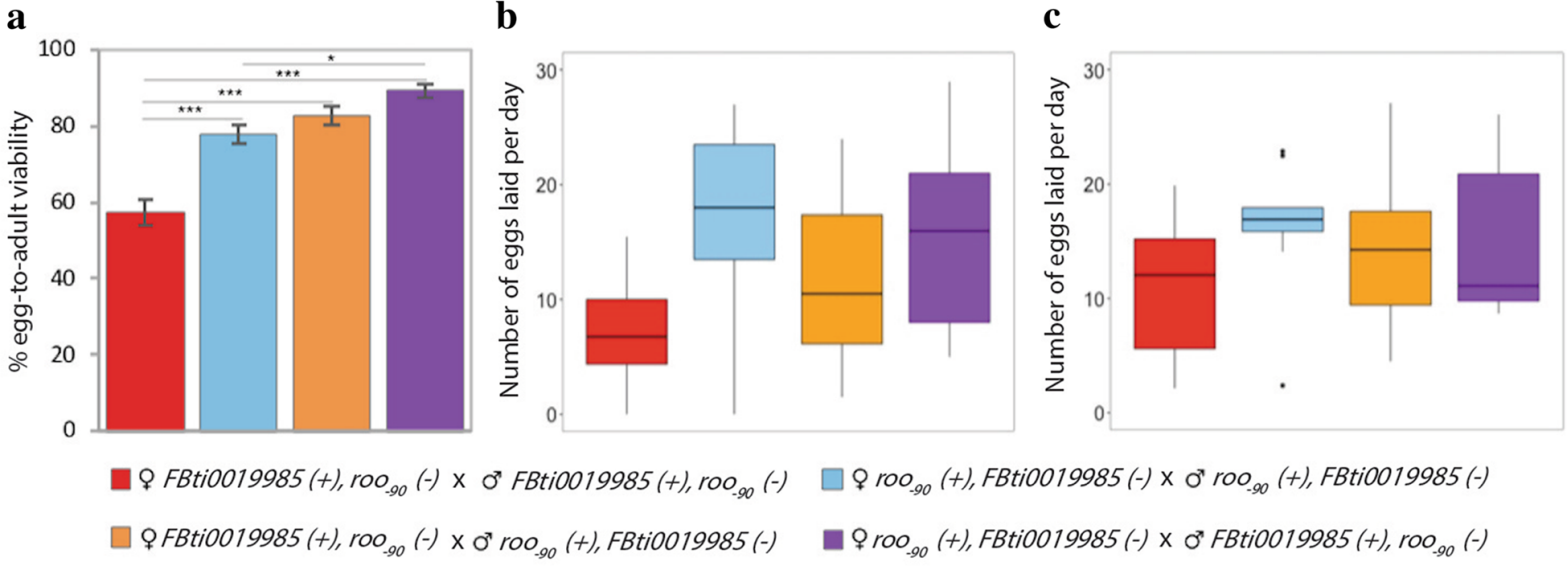
Egg-to-adult viability and fecundity are not likely to reduce the probability of finding flies with more than one *roo* element in the *CG18446* promoter region. **a**. Percentage of emerged flies from the four crosses tested. **b**. Average number of eggs laid per day during the first 48 h for each of the four crosses analyzed. **c**. Average number of eggs laid per day during 18 days for each of the four crosses analyzed

To discard that flies with two *roo* insertions have reduced egg-to-adult viability or reduced fecundity compared with flies containing only one *roo* insertion, we created flies containing two insertions in the *CG18446* promoter region (see Material and Methods). We found that flies with two *roo* insertions had similar or significantly higher viability compared with flies with only one of the *roo* insertions (ANOVA *p-value* < 0.001 Fig. 4a). Early fecundity of flies containing two *roo* insertions was not significantly different from that of flies containing only one *roo* insertion (ANOVA *p-value* = 0.068, Fig. 4b). Similarly, we did not find differences in the average number of eggs laid per day during 18 days between flies with one or two *roo* insertions (ANOVA *p-value* = 0.494, Fig. 4c). Note that the genetic background of flies containing one or two *roo* insertions is different. Thus, polymorphisms other than the presence/absence of these insertions are likely to be also contributing to the lack of differences observed.

## Discussion

Besides the nine *roo* solo-LTRs found in Merenciano et al. (2016) [10], we have discovered 11 new *roo* insertions in the *CG18446* promoter region. It is known that *D. melanogaster* populations differ in their TE content [10, 28–31]. Thus, it could be that analyzing more populations, especially from geographical areas currently underrepresented such as Central and South America or Asia, could lead to the discovery of more *roo* insertions in the *CG18446* promoter region. However, the number of populations analyzed in this work was seven-fold higher than in Merenciano et al. (2016) [10] and the number of new *roo* insertions was only twice that of our previous study, suggesting that it is likely that we have discovered the majority of the *roo* elements in the *CG18446* promoter region.

All 20 *roo* insertions identified in the promoter region of *CG18446* are solo LTR insertions, while the majority (21 out of 26) of the *roo* insertions found in other promoter regions are full-length insertions (Additional file 6 A). Solo LTRs are presumably the result of homologous unequal recombination between the two LTRs of a full-length element [14]. Thus, the recombination region where these TEs are located could influence whether they are full-length elements or solo LTRs. However, only four of the 21 full-length elements are present in regions with a low recombination rate, while the other 17 *roo* insertions are located in regions with a similar recombination rate as the *CG18446* promoter region (Additional file 6A) [32, 33]. Although it is not clear why all the insertions in the *CG18446* promoter region are solo LTRs, the location of this promoter in an open chromatin region could be one of the contributing factors as it has been suggested that chromatin accessibility favors double strand breaks and thus recombination [33].

Phylogenetic analyses revealed that the presence of multiple *roo* insertions in the *CG18446* promoter is likely to be the result of several bursts of transposition rather than small rearrangements or insertion preference for 5′ gene flanking regions. This is consistent with previous data suggesting that *roo* is one of the most active TE families with a high transposition rate [14, 15, 23, 24]. Indeed, it has been suggested that *roo* elements have been able to evade piRNA silencing, because the number of novel *roo* insertions is high despite the presence of a high proportion of piRNAs against this family [15]. Note that the piRNA data analyzed in Rahman et al. (2015) [15] was obtained from ovaries and ovarian cell cultures [34, 35], and it has been suggested that TE activity in female and male germlines might differ due to polymorphisms in the piRNA regulatory genes between sexes [36].

Why *roo* insertions recurrently insert in the promoter region of the *CG18446* gene is not yet completely understood. We showed that there is no other cluster of *roo* insertions in promoter regions or in 1 kb genomic regions genome-wide. Thus, the presence of multiple *roo* insertion in this particular promoter region is probably related to some specific feature of this promoter. We indeed found that chromatin accessibility could be one of the factors explaining the recurrent insertions in this promoter region. In *D. melanogaster*, one other insertional cluster described is also located in the promoter region of stress response genes, which is located in an open chromatin region [8]. Several of the TEs located in the promoter of *hsp* genes have been shown to affect the expression of the nearby genes by altering the promoter architecture [7, 37]. So far, only one of the *roo* insertions in the *CG18446* promoter region, *FBti0019985*, has been shown to affect the expression of this gene by adding a new transcription start site [10]. In this work, we found that *roo*_*− 90*_ has an allelic frequency of 16.5% and is significantly more frequent in arid climates. Thus, it would be interesting to test whether this insertion affects expression of the nearby gene and/or is associated with a fitness-related trait that could explain its higher frequencies in arid climate conditions.

Finally, in *Arabidopsis thaliana* recurrent insertion of TEs from the *Copia* family in the first intron of the *FLC* locus have been associated with epigenetic regulation of this locus in response to cold [38]. Thus, not only in *D. melanogaster* but at least also in *A. thaliana*, recurrent insertions of TEs belonging to a single family are associated with stress-related genes, and some of these insertions have fitness-related consequences.

## Material and methods

### Fly stocks

Fly stocks used for PCR screening are listed in Additional file 2A. One outbred population homozygous for the presence of *FBti0019985*, and one outbred population homozygous for the presence of *roo*_*− 90*_ were generated by a round-robin cross of inbred lines from the Drosophila Genetic Reference Panel (DGRP) [39] and isofemale lines from different European populations (Additional file 9). We maintained the population by random mating with a large population size for over five generations before starting the experiments. All flies were reared on fly food medium in a 12:12 h light/dark cycle at 25 °C.

### Analysis of TE presence/ absence

We used the same PCR approach as in Merenciano et al. (2016) [10] to check for the presence/absence of TE insertions in the *CG18446* promoter region in 234 natural strains from Europe, North America (DGRP) [16] and Africa (Nexus) [17]. Briefly, genomic DNA was extracted from a pool of 10 female flies of each strain. We performed PCR with two primer pairs. Primer pair Flanking (FL6) (5′-AACAATGCAAGTCCGTGCTC-3′) and Right (R) (5′-CGTAGGATCAGTGGGTGAAAATG-3′) are expected to give an 802 bp band when insertions are absent and a bigger band when there is an insertion. Primer pair Left (L) (5′ -AGTCCCTTAGTGGGAGACCACAG-3′) and R are expected to give a band only when there is a *roo* insertion. When the two PCRs failed, we used the alternative primer R2 (5′-CGGGTACATCTTTGCGGGAT-3′). When the PCR using the FL6 primer failed, we used the alternative primers FL (5′-GGCATCATAAAACCGTTGAACAC-3′), and/or FL7 (5′- TTCGTGCGTGTTCGGTACTT-3′). PCR products were purified using the NucleoSpin® Gel and PCR Clean-up kit (Macherey-Nagel) using the manufacturer’s instructions and Sanger-sequenced using FL and/or L and R primers to verify the results. PCR failed for 16 strains and thus we could analyze 218 out of the 234.

### Consensus motifs

We aligned using Genious 9.1.4 (https://www.geneious.com) the *roo* element sequences from the 114 strains that were fully sequenced in this work. We also included in the alignments the *roo* sequences reported in Merenciano et al. (2016) [10]. We identified in these sequences the nine transcription factor binding sites, the *Inr* promoter motif, and the MARs previously identified by Merenciano et al. (2016) [10]. We constructed the consensus sequence logos using WebLogo [40]. The target site duplication (TSD) consensus was constructed also using Weblogo with 15 out of 17 of the TSDs found in this work and in Merenciano et al. (2016) [10]. The two TSD removed have shorter sequence length.

### Phylogenetic analysis

We followed the same approach as in Merenciano et al. (2016) [10]. Briefly, 16 of the 20 *roo* solo-LTR insertions in *CG18446* promoter region were sequenced in several strains (Additional file 2A). For each of these 16 insertions, we aligned the sequences and generated a consensus. We then aligned these 16 consensus sequences, the other four *roo* insertions and the 115 *roo* insertions found in *D. melanogaster* genome using the multiple sequence aligner program MAFFT. We inferred a maximum likelihood tree under the general time-reversible nucleotide model and a gamma distribution of evolutionary rates, using RAxML Version 8 [41] (Additional file 10). We removed from the phylogenetic analysis those TEs with exact identical sequences. The interactive tree of life (iTOL) framework (https://itol.embl.de/) was used for the analysis and visualization of the tree, ignoring branch lengths.

### Analysis of other *roo* clusters in promoter regions

We analyzed the region where 27 *roo* elements are inserted less than 1 kb from a gene or in 5’UTR regions in the *D. melanogaster* reference genome (R6.07) in 10 randomly chosen DGRP strains. To determine if 10 strains are enough to detect a cluster, we ran 1000 randomly generated trials using a Python script. This script randomly chose 10 strains among all the DGRP strains screened by PCR in this work and in Merenciano et al. (2016) [10] and counted the number of different *roo* insertions obtained in every iteration. We found that four was the average number of different *roo* insertions that can be found in a screening of 10 randomly chosen DGRP strains. Then, by checking 10 different DGRP strains we expected to find an average of four different *roo* insertions in the case of the presence of an insertion cluster similar to the one found in the *CG18446* promoter region.

For each strain, genomic DNA was extracted from a pool of 10 female flies. Primers (forward and reverse) were design in the flanking region of the insertion amplifying a minimum of 500 bp when the TE is not present (Additional file 11). We also used a combination of primers (*roo*_primer and reverse) that gave a PCR band only when a *roo* element is present (Additional file 11). PCR programs were set according to the length of each TE insertion. In addition, we also considered *de novo* insertions found with TIDAL software in a set of 177 DGRP strains [15]. We first selected all the 559 *roo* elements predicted to be inserted less than 1 kb from the nearest gene or in 5’UTR regions. Then, we grouped the insertions based on the promoter region where they are inserted. Finally, we analyzed by PCR the 27 promoter regions where three or more strains putatively have a *roo* insertion. As before, genomic DNA was extracted from a pool of 10 female flies of each strain. Five combinations of primer pairs were used in order to verify the position of the insertion: one primer pair in the flanking region of the insertion amplifying a minimum of 500 bp when the TE is not present (ClusterF and ClusterR), and other four combinations where one primer was located in the LTR region in both genomic orientations (ClusterF and rooL2, ClusterR and rooL, ClusterF and rooL, and ClusterR and rooL2) (Additional file 11). PCR products were purified and Sanger sequenced as mentioned before. For the four promoter regions for which we found two *roo* insertions, 8, 13, 17, and 23, we performed additional PCRs following the same approach in ten DGRP strains (RAL-105, RAL-129, RAL-136, RAL-161, RAL-208, RAL-239, RAL-208, RAL-239, RAL-280, RAL-301, RAL-309, and RAL-379).

### Analysis of other clusters in the genome

We selected the 12,745 *roo* *de novo* insertions predicted by TIDAL software in 177 DGRP strains [15]. Since TIDAL software predicts a range of coordinates where the TEs may be inserted, we established as the insertion site the midpoint of the coordinates. For each chromosome arm (except the Y chromosome), we first considered as the same insertion those inserted within 5 bp windows. Thus, we got a total of 9243 *roo* *de novo* insertions. After that, we counted how many predicted *de novo* *roo* elements are in windows of 1 kb. We then chose for PCR validation five 1 kb regions with more than seven predicted *roo* insertions, and two additional 1 kb regions with four and six insertions predicted. Every region was validated in 7–10 different DGRP strains. For each strain, genomic DNA was extracted from a pool of 10 female flies. Five combinations of primers were designed following the same approach as before (Additional file 11). PCR products were purified and Sanger sequenced as mentioned before.

### Expected genotype frequency calculation

For all the populations analyzed in this work, the expected genotype frequencies of flies containing both *FBti0019985* and *roo*_*− 90*_ insertions were calculated multiplying the observed allelic frequency for *FBti0019985* and the observed allelic frequency for *roo*_*− 90*_*,* considering that they are in different haplotypes (Additional file 2E).

### Viability assays

We checked the egg-to-adult viability of outbred *FBti0019985* (+) crosses, outbred *roo*_*− 90*_ (+) crosses and their reciprocal crosses. In total, 100 five to seven day-old flies (50 males and 50 virgin females) for each cross were allowed to lay eggs for 24 h on apple juice-agar medium with fresh yeast at 25 °C. Embryos were collected following the protocol described in Schou et al. (2013) [42]. For each cross, we collected a total number of 150 embryos and put them in groups of 30 in empty vials with fresh food. We maintained the vials at 25 °C until adult emergence. The percentage of egg-to-adult viability was calculated as the ratio of the number of emerged flies to the total number of embryos placed in each vial. Statistical significance was calculated performing ANOVA using SPSS v.21 followed by Tukey post-hoc multiple comparison procedure.

### Fecundity assays

We checked the fecundity of outbred *FBti0019985* (+) crosses, outbred *roo*_*− 90*_ (+) crosses and their reciprocal crosses. For each cross, 10 virgin females were placed individually with one male in vials with fresh food. Flies were moved to new vials every day during 18 days without CO_2_ anesthesia, and dead males were replaced. The number of eggs laid per day was counted every day during this period. The average of the total number of eggs laid per day during the 18 days (total fecundity), and the average of the total number of eggs laid per day during the first 48 h (early fecundity) was compared between crosses. We removed from the analysis those vials where the female died during the experiment. Statistical significance was calculated performing ANOVA using SPSS v.21.

## Additional files

Additional file 1: Populations used for the analysis. (XLSX 11 kb)

Additional file 2: **A.** PCR results for the 277 strains analyzed in this work and in Merenciano et al. (2016). Strains used in Merenciano et al.* (*2016) are highlighted in blue. **B.** *Tlex-2* predictions in Merenciano et al. (2016) compared to PCR results in this work. Correct predictions are highlighted in green. Strains with *roo* insertions not identified in Merenciano et al. (2016) are highlighted in orange. Strains for which no results were obtained either by *T-lex2* or by PCR are highlighted in grey. **C.** Allelic frequencies of all the 20 *roo* insertions in all the populations analyzed. EU: Europe, NA: North America and ZI: Zambia. **D.** Allelic frequencies (%) of the 20 *roo* insertions in the 15 different populations analyzed. Elements only present in one population are highlighted in red. **E.** Expected genotype frequency of heterozygous flies with the two most common insertions, *FBti0019985* and *roo-90* in all the populations analyzed. a: *FBti0019985* alellic frequency, b: *roo-90* alellic frequency, and c: absent alellic frequency. (XLSX 47 kb)

Additional file 3: **A.** Consensus target site duplication (TSD) sequence identified in Merenciano et al. (2016) (left panel) and consensus TSD identified with the data of this paper and Merenciano et al. (2016) (right panel). **B.** TSD sequences of the 20 *roo* insertions. Frequency represents the number of strains that harbor the TSD out of the number of strains with a complete sequenced region. (DOCX 332 kb)

Additional file 4: Consensus sequence of the transcription factor binding sites and matrix attachment regions identified in all the *roo* sequences identified in the *CG18446* promoter region. (DOCX 330 kb)

Additional file 5: The formation of *roo* insertional clusters in gene promoter regions is not a *roo* family characteristic. Scheme of the gene promoter regions containing *roo* elements present in the reference genome (left) and present in 177 DGRP inbred strains (right). (DOCX 29 kb)

Additional file 6: **A.** Coordinates (R6), length, recombination rates and PCR results of the 26 promoter regions with a *roo* insertion in the reference genome. **B.** PCR results and *de novo* TE information of the 28 promoter regions where > = 3 strains putatively have a roo insertion based on TIDAL software predictions. **C.** Promoter regions where < 3 strains putatively have a *roo* insertion based on TIDAL software predictions. (XLSX 61 kb)

Additional file 7: Genome-wide distribution of *de novo* *roo* elements found in 177 DGRP strains. Number of predicted *de novo* *roo* elements found in 177 DGRP strains inserted in 1 kb windows in chromosomes 2, 3, 4, and X. (DOCX 102 kb)

Additional file 8: **A.** PCR results of the five 1 kb regions with more *roo* insertions predicted by TIDAL software. **B.** 1 kb regions with at least 1 *roo* insertion predicted by TIDAL software. Regions checked by PCR are highlighted in yellow. (XLSX 166 kb)

Additional file 9: Schematic representation of the round-robin cross-design for outbred *FBti0019985* (+), *FBti0019985* (−), *roo*_*− 90*_ (+), and *roo*_*− 90*_ (−) generation. (DOCX 123 kb)

Additional file 10: Phylogenetic tree of the 20 *roo* solo-LTR found in *CG18446* promoter region and 115 other *roo* insertions annotated in the reference genome. (TXT 6 kb)

Additional file 11: List of primers used for insertional cluster validation. (XLSX 12 kb)

## Abbreviations

DGRP: Drosophila Genetic Reference Panel
LTR: Long Terminal Repeat
TE: Transposable Element
TSD: Target Site Duplication
