# Supplementary material for: A unique cluster of roo insertions in the promoter region of a stress response gene in Drosophila melanogaster
Source: Mob DNA. 2019 Mar 13;10:10. doi: 10.1186/s13100-019-0152-9 (PMC6415491; doi:10.1186/s13100-019-0152-9)
Supplement: Supplementary file 3 — A. Consensus target site duplication (TSD) sequence identified in Merenciano et al. (2016) (left panel) and consensus TSD identified with the data of this paper and Merenciano et al. (2016) (right panel). B. TSD sequences of the 20 roo insertions. Frequency represents the number of strains that harbor the TSD out of the number of strains with a complete sequenced region. (DOCX 332 kb) [file 13100_2019_152_MOESM3_ESM.docx]

**Additional file 3**

**
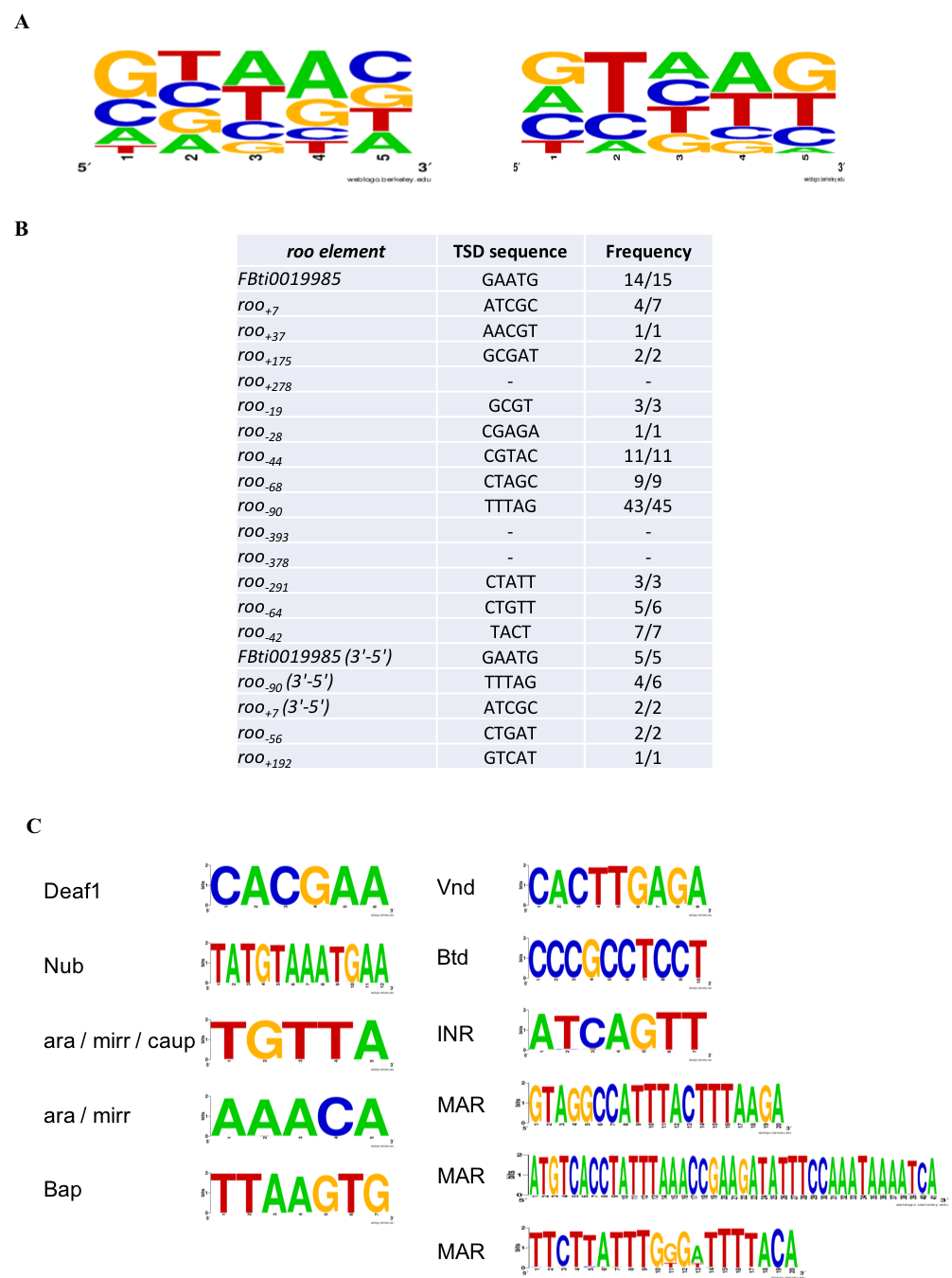
**

**B**

| *roo* element | TSD sequence | Frequency |
| --- | --- | --- |
| *FBti0019985* | GAATG | 14/15 |
| *roo_+7_* | ATCGC | 4/7 |
| *roo_+37_* | AACGT | 1/1 |
| *roo_+175_* | GCGAT | 2/2 |
| *roo_+278_* | ***-*** | - |
| *roo_-19_* | GCGT | 3/3 |
| *roo_-28_* | CGAGA | 1/1 |
| *roo_-44_* | CGTAC | 11/11 |
| *roo_-68_* | CTAGC | 9/9 |
| *roo_-90_* | TTTAG | 43/45 |
| *roo_-393_* | - | - |
| *roo_-378_* | - | - |
| *roo_-291_* | CTATT | 3/3 |
| *roo_-64_* | CTGTT | 5/6 |
| *roo_-42_* | TACT | 7/7 |
| *FBti0019985 (3’-5’)* | GAATG | 5/5 |
| *roo_-90_ (3’-5’)* | TTTAG | 4/6 |
| *roo_+7_ (3’-5’)* | ATCGC | 2/2 |
| *roo_-56_* | CTGAT | 2/2 |
| *roo_+192_* | GTCAT | 1/1 |

**Additional file 3. A**. Consensus target site duplication (TSD) sequence identified in Merenciano et al. (2016) (left panel) and consensus TSD identified with the data of this paper and Merenciano et al. (2016) (right panel). **B.** TSD sequences of the 20 *roo* insertions. Frequency represents the number of strains that harbor the TSD out of the number of strains with a complete sequenced region.
