# Supplementary material for: A unique cluster of roo insertions in the promoter region of a stress response gene in Drosophila melanogaster
Source: Mob DNA. 2019 Mar 13;10:10. doi: 10.1186/s13100-019-0152-9 (PMC6415491; doi:10.1186/s13100-019-0152-9)
Supplement: Supplementary file 4 — Consensus sequence of the transcription factor binding sites and matrix attachment regions identified in all the roo sequences identified in the CG18446 promoter region. (DOCX 330 kb) [file 13100_2019_152_MOESM4_ESM.docx]

**Additional file 4**


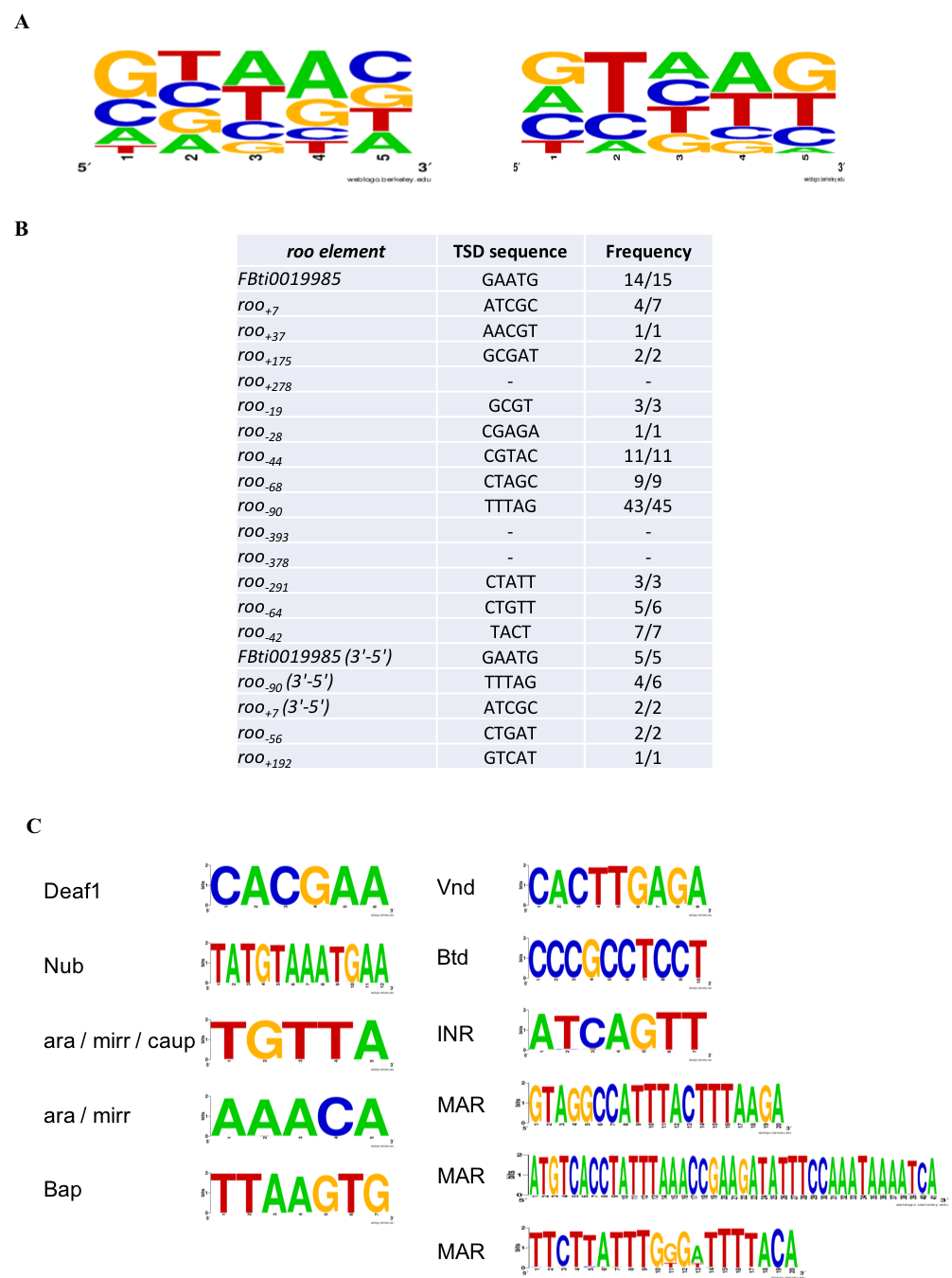


**Additional file 4**. Consensus sequence of the transcription factor binding sites and matrix attachment regions identified in all the *roo* sequences identified in the *CG18446* promoter region.
