# Supplementary material for: A unique cluster of roo insertions in the promoter region of a stress response gene in Drosophila melanogaster
Source: Mob DNA. 2019 Mar 13;10:10. doi: 10.1186/s13100-019-0152-9 (PMC6415491; doi:10.1186/s13100-019-0152-9)
Supplement: Supplementary file 5 — The formation of roo insertional clusters in gene promoter regions is not a roo family characteristic. Scheme of the gene promoter regions containing roo elements present in the reference genome (left) and present in 177 DGRP inbred strains (right). (DOCX 29 kb) [file 13100_2019_152_MOESM5_ESM.docx]

**Additional file 5**

>= 3 *roo* / region

<1kb gene or 5’UTR

Reference genome

138 *roo*

27 *roo*

12,745 *roo*

559 *roo*

27 gene promoter regions

28 gene promoter regions

177 DGRP inbred strains

1 gene promoter region with 20 *roo* (***CG18446***)

26 gene promoter regions with one *roo*

1 gene promoter region with 20 *roo* (***CG18446***)

23 gene promoter regions with one *roo*

4 gene promoter regions with two *roo*

**Additional file 5. The formation of *roo* insertional clusters in gene promoter regions is not a *roo* family characteristic.** Scheme of the gene promoter regions containing *roo* elements present in the reference genome (left) and present in 177 DGRP inbred strains (right).
