# Supplementary material for: A unique cluster of roo insertions in the promoter region of a stress response gene in Drosophila melanogaster
Source: Mob DNA. 2019 Mar 13;10:10. doi: 10.1186/s13100-019-0152-9 (PMC6415491; doi:10.1186/s13100-019-0152-9)
Supplement: Supplementary file 7 — Genome-wide distribution of de novo roo elements found in 177 DGRP strains. Number of predicted de novo roo elements found in 177 DGRP strains inserted in 1 kb windows in chromosomes 2, 3, 4, and X. (DOCX 102 kb) [file 13100_2019_152_MOESM7_ESM.docx]

**Additional file 7**

**
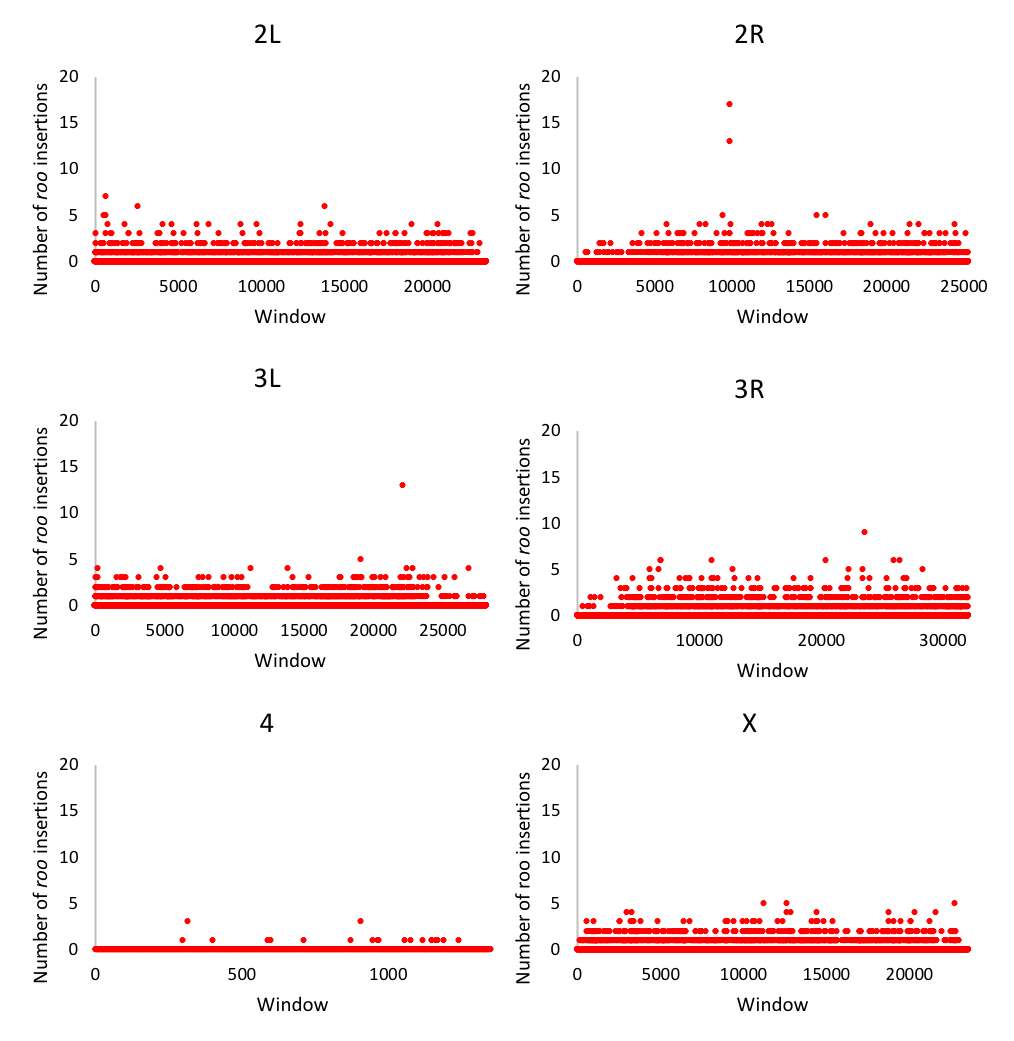
**

**Additional file 7. Genome-wide distribution of *de novo roo* elements found in 177 DGRP strains.** Number of predicted *de novo roo* elements found in 177 DGRP strains inserted in 1kb windows in chromosomes 2, 3, 4, and X.
