# Supplementary material for: A unique cluster of roo insertions in the promoter region of a stress response gene in Drosophila melanogaster
Source: Mob DNA. 2019 Mar 13;10:10. doi: 10.1186/s13100-019-0152-9 (PMC6415491; doi:10.1186/s13100-019-0152-9)
Supplement: Supplementary file 9 — Schematic representation of the round-robin cross-design for outbred FBti0019985 (+), FBti0019985 (−), roo− 90 (+), and roo− 90 (−) generation. (DOCX 123 kb) [file 13100_2019_152_MOESM9_ESM.docx]

**Additional file 9**

**
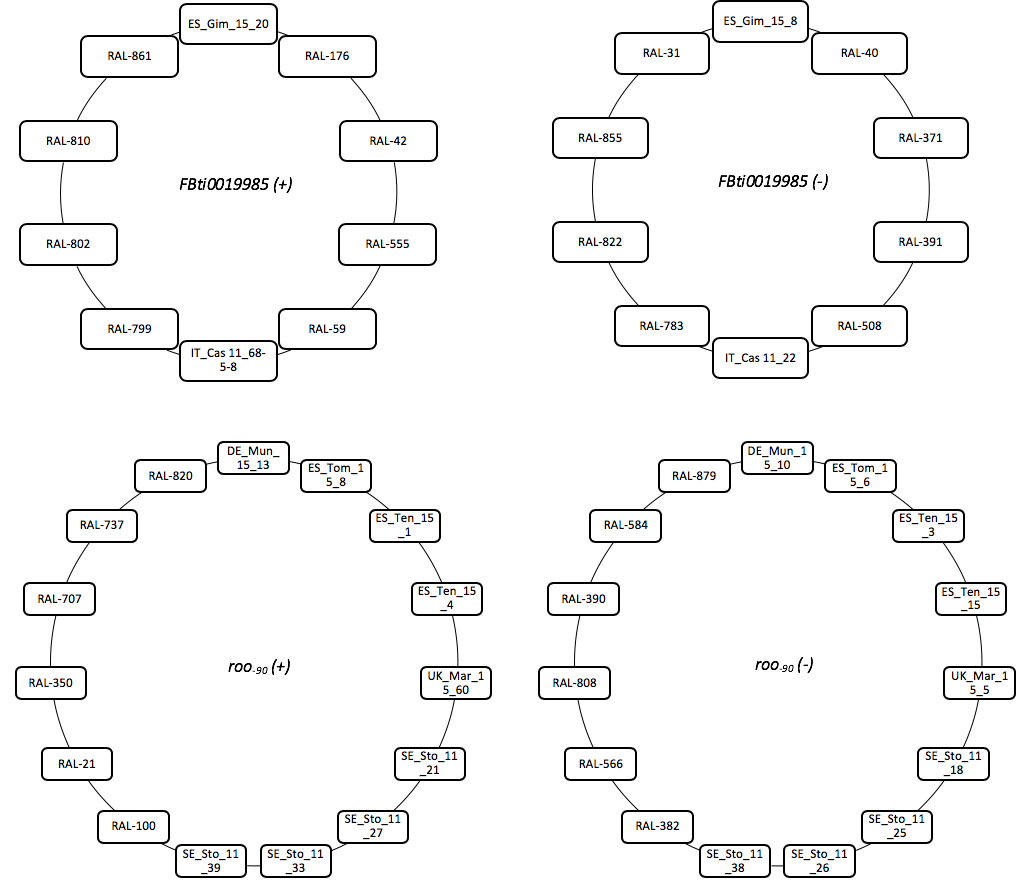
**

**Additional file 9.** Schematic representation of the round-robin cross-design for outbred *FBti0019985* (+), *FBti0019985* (-), *roo_-90_* (+), and *roo_-90_* (-) generation.
